# New findings on primary and acquired resistance to anti-EGFR therapy in metastatic colorectal cancer: do all roads lead to RAS?

**DOI:** 10.18632/oncotarget.4959

**Published:** 2015-07-22

**Authors:** Giuseppe Bronte, Nicola Silvestris, Marta Castiglia, Antonio Galvano, Francesco Passiglia, Giovanni Sortino, Giuseppe Cicero, Christian Rolfo, Marc Peeters, Viviana Bazan, Daniele Fanale, Antonio Giordano, Antonio Russo

**Affiliations:** ^1^ Department of Surgical, Oncological and Oral Sciences, University of Palermo, Palermo, Italy; ^2^ Medical Oncology Unit, National Cancer Institute “Giovanni Paolo II”, Bari, Italy; ^3^ Department of Oncology, University Hospital of Antwerp, Edegem, Belgium; ^4^ Sbarro Institute for Cancer Research and Molecular Medicine, Temple University, Philadelphia, PA, USA; ^5^ Department of Medicine, Surgery & Neuroscience, University of Siena, Siena, Italy

**Keywords:** RAS, colorectal cancer, epidermal growth factor receptor, cetuximab, panitumumab

## Abstract

Anti-epidermal growth factor receptor therapy with the monoclonal antibodies cetuximab and panitumumab is the main targeted treatment to combine with standard chemotherapy for metastatic colorectal cancer. Many clinical studies have shown the benefit of the addition of these agents for patients without mutations in the EGFR pathway. Many biomarkers, including KRAS and NRAS mutations, BRAF mutations, PIK3CA mutations, PTEN loss, AREG and EREG expression, and HER-2 amplification have already been identified to select responders to anti-EGFR agents. Among these alterations KRAS and NRAS mutations are currently recognized as the best predictive factors for primary resistance. Liquid biopsy, which helps to isolate circulating tumor DNA, is an innovative method to study both primary and acquired resistance to anti-EGFR monoclonal antibodies. However, high-sensitivity techniques should be used to enable the identification of a wide set of gene mutations related to resistance.

## INTRODUCTION

Colorectal cancer (CRC) is one of the leading causes of cancer-related death. Around half of CRC patients develop distant metastases, which can be detected early at diagnosis or at a later stage. Despite the development of different treatment options, the outcome for patients with unresectable metastatic lesions is still unfavorable and the metastatic spread to the liver is the major contributor to mortality in CRC. New drugs such as oxaliplatin, irinotecan, bevacizumab, aflibercept, cetuximab, panitumumab and regorafenib have led to a significant improvement in median survival from 6-7 months to 24-30 months in patients with unresectable metastatic CRC (mCRC), also improving quality of life [1, 2]. New biological agents mainly target two different pathways: tumor growth mediated by proangiogenic factors, and Epidermal Growth Factor Receptor (EGFR)-triggered cell proliferation [3]. Predictive biomarkers with clinical relevance for sensitivity to anti-angiogenic drugs have not yet been defined [4]. Conversely, anti-EGFR monoclonal antibodies (moAbs) are effective in those patients whose tumors do not have specific biomarkers, such as KRAS, NRAS, BRAF, and PIK3CA gene mutations [5, 6]. The establishment of the predictive role of these and other biomarkers has allowed the identification of patients suitable for anti-angiogenic therapy and spares others from unnecessary anti-EGFR therapy [7, 8].

The majority of these biomarkers are still under investigation to define the mechanisms of primary resistance. In the meantime some preclinical studies are evaluating the development of acquired resistance to anti-EGFR moAbs.

The new methodology of liquid biopsy has added new perspectives to the standard biomarker evaluation in tissue biopsy. The different sensitivity of various techniques is the main parameter to reach proper clinical validation. This review aims to highlight the recent status of translational research on mechanisms and predictive factors of intrinsic and acquired resistance.

## THE IMPORTANCE OF DETECTION METHODS

In recent years, new targeted therapies have been exploited in the treatment of mCRC patients. Cetuximab and Panitumumab are monoclonal antibodies that bind to EGFR, causing anti-tumor activity [9-12]. Nevertheless, these drugs show a clinical benefit in only a small percentage of cancer patients and this is mainly due to molecular alterations in EGFR pathway effectors [13]. It has been demonstrated in several randomized controlled trials that somatic single-nucleotide point mutations in codons 12 and 13 of the KRAS gene determine a constitutive activation of the MAPK pathway, thus leading to anti-EGFR treatment resistance. KRAS mutations are negative predictive markers and patient selection for anti-EGFR treatment is dependent on KRAS mutational status [12]. Recently it has been proved that the mutational status of other RAS family genes (specifically NRAS), but also KRAS mutations outside exon 2, are negative predictors for response to anti-EGFR drugs [14]. Furthermore, a recent meta-analysis demonstrated that approximately 20% of *KRAS* exon 2 wild-type tumors harbor one of the new *RAS* mutations. Thus CRC patients without any *RAS* mutations (either *KRAS* exon 2 or new *RAS* mutations) significantly benefit from anti-EGFR treatment compared with tumors with any of the new *RAS* mutations [15].

Given that patients diagnosed with mCRC are routinely tested for RAS mutational status, it is known that 35-50% of CRC harbor RAS mutations [16]. RAS testing has become mandatory for an appropriate treatment decision. There are several methodologies for RAS mutation detection with different advantages and disadvantages.

Despite the promising advances that have been made so far there is still a lack of a clinically-proven circulating biomarker that can be used to guide patient management [17]. To date, the follow-up of mCRC patients has mainly been based on imaging techniques (CT scan, PET, MRI etc…) and serum-based protein biomarkers such as the carcinoma antigen-125 (CA-125) and the carcinoembryonic antigen (CEA). The main limitation in using these biomarkers is that they are detectable also in healthy individuals (albeit at a lower concentration) and thus they do not perfectly fit one of the most important features of a circulating biomarker, i.e. to be highly specific for a pathological condition. The Cancer Genome Atlas (TCGA) is an international database that collects genomic information from cancer genome sequencing and it is contributing to expand knowledge about the molecular biology of several tumor types. It is noteworthy that almost every tumor type harbors somatic genetic alterations and that these somatic alterations arise prevalently in cancer cells. Therefore somatic alterations are 100% specific for cancer and can be used as specific biomarkers from a biological perspective [18]. Until some time ago the only source of tumor DNA was represented by a tumor tissue sample, but nowadays we know that DNA, from both healthy and cancer cells, is also diffused into the circulation. Therefore a relatively non-invasive source of tumor DNA is represented by plasma or serum. Cell-free circulating tumor DNA (ctDNA) is a promising candidate biomarker for the detection, monitoring and prognostic prediction of malignant tumors and can be defined also as a liquid biopsy [19, 20]. A liquid biopsy is a biomarker that can be easily isolated from blood and, as well as a tissue biopsy, is representative of the tissue from which it is spread [21].

### Tissue DNA *vs* circulating DNA

DNA mutational analysis on formalin-fixed paraffin embedded (FFPE) tissue is nowadays a routine practice in medical oncology. The identification of a specific tumor genotype is mandatory for treatment decision and RAS testing is always requested for mCRC patients.

Currently the evaluation of RAS mutational status from FFPE is the only procedure recognized and accepted in clinical practice. In the near future, we might witness the introduction of ctDNA testing as a precise, efficient and non-invasive technique for mutation detection both at diagnosis and during subsequent follow-up.

Circulating free DNA may also have a diagnostic value. Cell-free DNA (cfDNA) is released from both healthy and cancer cells. Healthy cells are normally destroyed through a regulated and orderly process; in apoptotic cells DNA is uniformly truncated into 185- to 200-bp fragments. CfDNA released by necrotic cells, and hence mainly tumor cells, varies in length. Thus a preponderance of longer DNA fragments could be a marker for tumor detection [22, 23]. The DNA integrity index can be calculated as the ratio of longer to shorter fragments. In 2006 Umetani et al. analyzed the DNA Integrity Index (DII) in colorectal or periampullary cancer [24]. The aim of the study was to evaluate ALU-repeats fragments of different lengths by means of quantitative PCR (qPCR). The short fragments (115bp) also represent the total amount of circulating DNA whereas the long fragments (247bp) are assumed to derive from necrotic cells. The ratio of ALU247/ALU115 is defined as the DII and was found to be significantly increased even in localized CRC and periampullary cancers. Recently T.B. Hao et al. also demonstrated that DII evaluation might be a useful biomarker for the diagnosis and prognostic prediction of colorectal cancer [25] and the combination with CEA could improve the diagnostic efficiency for CRC. CfDNA concentration and DII are interesting tools that might be valuable in the early complementary diagnosis and monitoring of progression and prognosis of CRC.

As previously mentioned, RAS testing in FFPE tissue is considered a routine practice but it is nonetheless challenging, mainly due to the DNA degradation that occurs during fixation procedures. The analysis of FFPE material gives a snapshot of the tumor in a specific moment but it does not provide any information about disease evolution or heterogeneity. ctDNA analysis offers the challenging opportunity to follow tumor molecular modifications over time and space. We can repeatedly assess ctDNA at different time-points during treatment. For metastatic patients, with multiple lesions, ctDNA is composed of a mixture of DNA released from the different lesions, and thus allows evaluation beyond the tumour tissue heterogeneity, even though we might not be able to detect exactly which lesions harbor a specific mutation.

It is known that KRAS mutations confer primary resistance to anti-EGFR treatment, but it is still not clear whether mutations in this gene are acquired after treatment or are pre-existing at low levels in tumors with ostensibly wild type KRAS genes. Diaz et al. in 2012 determined whether mutant KRAS DNA could be detected in the circulation of patients receiving monotherapy with anti-EGFR drugs [26]. 38% of patients, whose tumors were initially KRAS wild type, developed detectable mutations of KRAS in their ctDNA. The mutations might presumably arise in a small subclone population of cancer cells and then expand under the pressure of the EGFR blockade. Furthermore, Bettegowda et al. demonstrated for the first time that NRAS codon 61 mutations are involved in acquired resistance [17].

### Low- *vs* high-sensitivity techniques

Sanger sequencing was previously the gold standard methodology to detect KRAS mutations. This technique has the advantage of being relatively cheap; it is a reliable assay with good clinical applicability and has a well-defined track record [27]. Furthermore it allows the detection of all potential variations, including base substitutions, insertions and deletions. Nevertheless the biggest disadvantage of Sanger sequencing is its relatively low sensitivity. This technique is rarely sensitive to mutant allele frequencies of less than 10%, which correspond to the threshold of 20% of tumor cells heterozygous for a mutation [28]; it also requires a large amount of mutated DNA relative to the normal DNA present in the sample. Therefore in this case it is fundamental to carefully review the material to ensure high tumor content. Nonetheless, Sanger sequencing is still a useful technique for RAS mutation testing, and most laboratories use Sanger sequencing followed by pyrosequencing. This technique is becoming more commonly used because of its high analytical sensitivity, being able to detect less than 5% of a specific mutation in a background of wild type DNA.

Real-time PCR has been suggested as a reliable, feasible and low time-consuming method for RAS mutation testing. In addition the technique shows small intra- and inter-lot deviations and a good concordance among the different real- time PCR systems [29].

**Figure 1:**
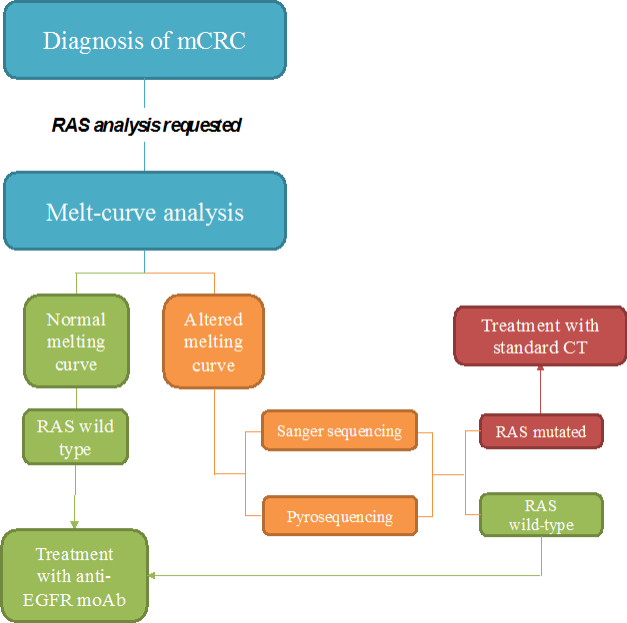
Proposed flow-chart of RAS analysis with Melting curve test After the diagnosis of metastatic colorectal cancer, RAS analysis is always requested before treatment initiation. Melt-curve analysis allows a quick screening of all RAS mutations. If the melting curve has a normal profile the sample can be reported as wild type and thus the patient can be selected for the specific targeted treatments. If the melting curve is altered it is mandatory to proceed with other tests (either Sanger sequencing or pyrosequencing), in order to identify the mutation. If a mutation is detected the patient is subjected to standard chemotherapy (CT); in the opposite case (RAS wild type) patients are selected for anti-EGFR treatment.

Real-time PCR methods are mainly based on two principles: melt-curve and allele-specific PCR analysis. The former allows obtainment of a quick screening of the possible genetic variants present in the samples. The melt-curve analysis is based on the use of fluorescent probes complementary to the target amplicon and it evaluates the differences in the melting temperature needed to dissociate probe from target [30]. Differences in melting temperature are detected based on the loss of fluorescence as a function of increasing temperature (Figure 1).

Conversely, allele-specific PCR allows the identification of a specific mutation. It is based on the use of oligonucleotide primers that specifically amplify mutant versus wild-type sequences through the differential binding and extension of the primer sequences to the target template. A particular application of allele-specific PCR is the so-called CAST-PCR (Competitive Allele-Specific TaqMan PCR). Each mutant allele assay detects specific or multiple mutant alleles and it is made up of: an allele-specific primer that detects the mutant allele; an oligonucleotide blocker that suppresses the wild type allele; a locus specific primer; and a locus specific TaqMan probe. This peculiar assay design allows the exquisite amplification of the mutant allele thus increasing the sensitivity of the technique.

**Figure 2:**
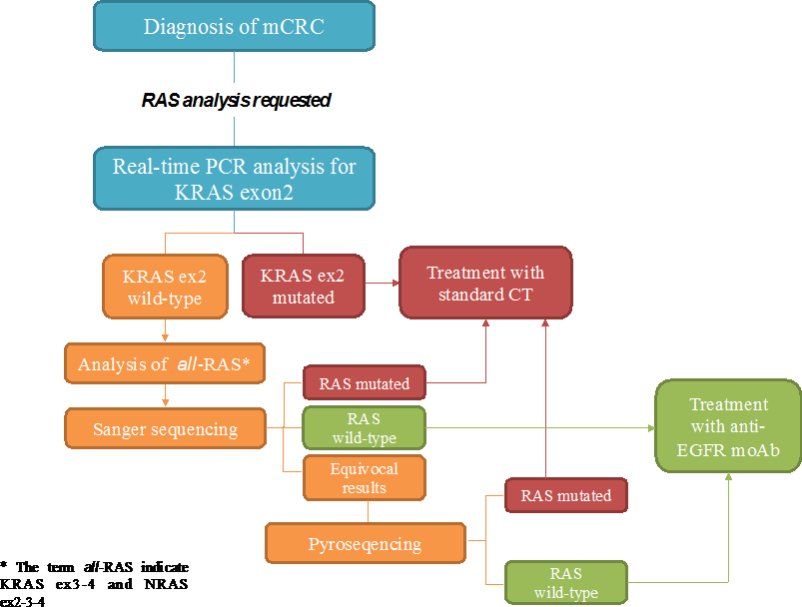
Proposed flow-chart of RAS analysis with the combination of different techniques After the diagnosis of mCRC, firstly KRAS exon 2 (codon12-13) can be tested through allele-specific Real-Time PCR. This has a strong rationale since the majority of RAS mutations fall into these codons. When a mutation is detected the patient is subjected to standard chemotherapy (CT). If no mutations are detected it is requested to proceed with the analysis of KRAS exon3-4 and NRAS exon2-3-4 (*all*-RAS) through Sanger sequencing. The results from this analysis may be: RAS mutated (standard CT); RAS wild type (target treatment), equivocal results. In the last case it is advisable to proceed with pyrosequencing.

Due to the differences in terms of performance between the various techniques, it may be recommended to combine different assays in order to provide the best means to assess clinical samples, particularly those that have low tumor content or from which DNA quality may not be optimal (Figure 2, Table 1).

**Table 1:** List of the main techniques used for detecting gene mutations

METHOD	SENSITIVITY		ADVANTAGE	DISADVANTAGE
	*Low*	*High*		
Sanger Sequencing	X		Ability to identify all mutations (also rare and unknown) Low costs	It requires a large amount of mutated DNA relative to the wild-type DNA Carefully review the material to ensure high tumor content Low sensitivity, 20% High time-consuming process
Pyrosequencing		X	It detects less than 5% of a specific mutation in a background of wild type DNA It requires low amount of starting material Low-time consuming process (results within 4 hours)	High costs Needs of dedicated instrument, reagents and plastics Problems in results interpretation
Melt-curve	X		Fast screening methods for all mutations Low-time consuming process Low costs	Problems in results interpretation Carefully review the material to ensure high tumor content In case of suspected mutations further analysis are required to identify the variant
Competitive Allele-Specific TaqMan PCR (CAST-PCR)		X	It detects as low as 0.1% - 1% mutated DNA in a background wild-type DNA Low-time consuming process (results within 3 hours)	Only known mutations can be detected, rare or unknown variants may be missed
Amplification Refractory Mutation System (ARMS)		X	Some commercial kits are CE-marked It detects 1% of a specific mutation in a background of wild type DNA	High costs

Nowadays there is also a series of kits developed by *in vitro* diagnostic (IVD) manufacturers both for research purposes and for use in clinical diagnostics. For instance the TheraScreen^®^ assay (DxS, Manchester, UK), based on the amplification refractory mutation system (ARMS) and Scorpion probes, is a CE-marked kit that allows the detection and qualitative assessment of KRAS gene mutations. Interestingly, the Food and Drug Administration (FDA) has approved the TheraScreen^®^ KRAS RGQ PCR Kit (Qiagen, Manchester, UK) to be applied as a companion diagnostic test for Cetuximab (Erbitux). This kit uses the Scorpions^®^ and ARMS^®^ technologies and allows the detection of six mutations in codon 12 and one in codon 13 of KRAS [31, 32]. Nevertheless the TheraScreen^®^ kit does not detect mutations in codon 61 of KRAS.

## CLINICAL APPLICATIONS OF RAS-RELATED PRIMARY RESISTANCE

Initial assessment of RAS mutational status, known as extended assessment exons, is the key to be able to define the best possible treatment for each mCRC patient. In particular, it aids in making the right choice of first-line chemotherapy regimen, since the presence of a possible mutation allows identification of a group of patients who would not obtain any benefit from the use of an anti-EGFR monoclonal antibody (moAb), cetuximab or panitumumab [2, 13, 33, 34].

This is to be considered as a fundamental step that will affect subsequent lines of treatment and, ultimately, progression free survival (PFS) and overall survival (OS) of our patients. Of note, the need to obtain a rapid reduction of the tumor mass represents a key point that influences the choice of chemotherapy regimen. For these reasons, clinical research through the study of molecular genetics has produced numerous efforts in search of predictors of response to various treatments. To date, studies have produced evidence sufficient only as regards RAS mutations, initially regarding codons 12 and 13 of exon 2 of the KRAS gene. Regarding the G13D mutation, 16% of KRAS mutations, there are however conflicting data [35, 36].

More recently the retrospective analysis of the prospective phase III PRIME study, which randomized KRAS wild type mCRC patients to receive first line FOLFOX + panitumumab vs FOLFOX, demonstrated the negative predictive value of mutations in exons 3 and 4 of KRAS and 2, 3, 4 of NRAS. Indeed, in KRAS mutant patients the addition of panitumumab to FOLFOX seems to have a detrimental effect on PFS (HR = 1:31; 95% CI: 1.07-1.60) and OS (HR = 1:21; 95% CI: 1:01 to 1:45). Conversely, median OS was 25.8 mo vs. 20.2 mo (HR = 0.77; 95% CI: 0.64-0.94, P = 0.009) in all RAS wild-type populations in favour of the combination of panitumumab and FOLFOX [15, 37].

Similar results were obtained from the retrospective analysis including extended RAS evaluation carried out on phase III CRYSTAL and phase II OPUS studies, which randomized patients receiving first-line cetuximab in combination with FOLFIRI or FOLFOX respectively. In the CRYSTAL study no benefit was observed for the experimental arm (FOLFIRI + Cetuximab) compared to FOLFIRI alone in terms of RR, PFS and OS in the RAS mutant subgroup, while the same analysis on all RAS wild type patients showed an increase of 27.7% in response rate (RR), an increase of about 3.0 months in median PFS, and an increase of 8.2 months in OS [38, 39].

In the OPUS study, patients with any *RAS* mutation (KRAS exon 2 + new RAS) received no benefit from the addition of cetuximab to FOLFOX4; in some cases, the addition of cetuximab showed a statistically non-significant trend to a worse outcome (ORR in RAS-mut patients to FOLFOX4 + cetuximab 37% and to FOLFOX4 alone 51%; *P* = .087). No statistically significant differences in median overall survival were observed in patients with KRAS-WT or RAS-mut tumors when cetuximab was added to FOLFOX4 [40].

The results of these first-line studies (CRYSTAL, PRIME and OPUS), comprising the extended panel of RAS mutations, have also demonstrated with greater conviction the effectiveness of the addition of anti-EGFR moAbs to, a standard chemotherapy regimen in terms of PFS (11.4, 12.0, 10.1 vs. 8.4, 5.8, 7.9 months, respectively) and in terms of OS (28.4, 19.8, 26.0 vs 20.2, 17.8, 20.2 months, respectively). The unique role as a negative predictor of response to anti-EGFR molecules of RAS mutations has been further strengthened by the final results of the TRIBE study, a prospective phase III trial which randomly assigned patients, never treated for advanced disease, to receive FOLFIRI + Bevacizumab or FOLFOXIRI + Bevacizumab. The subgroup analysis extended to not frequent RAS mutations showed in RAS and BRAF wild-type patients a trend in favor of the triplet-based regimen in terms of PFS (13.3 vs 11.3 months) and in terms of OS (41.7 vs 34.4 months). Of note, in the RAS mutant population the results were very similar (12.0 PFS, OS 28.6 months) between the two treatment arms, confirming the negative predictive role of RAS mutations. On the contrary, these results seem to confirm the data on the role of negative prognostic BRAF mutations (BRAF wild type and mut OS 41.7 and 19.1 months, respectively, in the arm containing the triplet) [41, 42].

In spite of the confirmed negative prognostic role of BRAF mutations (8% of RAS wild-type), their role as predictive of response to an anti-EGFR based regimen has not yet been defined because of discordant outcomes from frontline studies (CRYSTAL, PRIME and OPUS) and other studies.

Expectations were high regarding the results from two studies that compared head to head a first-line treatment consisting of a chemotherapeutic doublet with the addition of anti-EGFR or anti-VEGF mAbs. The FIRE-3study, a phase III trial, compared FOLFIRI + bevacizumab to FOLFIRI + cetuximab in a population of KRAS exon 2 wild-type patients. In spite of a difference in terms of RR, the primary endpoint of the study was not highlighted in the primary analysis, and the retrospective analysis extended to rare RAS mutations showed substantial differences in terms of RR (72% vs 56.1%) in the cetuximab arm (p = 0.003). As regards early tumor shrinkage (ETS), PFS in patients with ETS in the cetuximab arm was 9.7 months vs 5.8 months in patients with no-ETS. In the bevacizumab arm the PFS in ETS patients was 11.7 months vs 8.3 months in non-ETS patients, and finally deepness of response (DpR) correlated significantly with OS and PFS (p = 0.0003 in KRAS exon 2 wild-type patients and p < 0.0001 in the final RAS wild type population).

These important results were partially confirmed by the data from the second phase III study, the CALGB 80405 trial, comparing patients for the KRAS wild-type exon 2 subjected to a combination regimen at the discretion of the investigator (FOLFOX or FOLFIRI) with the addition of an anti EGFR or anti-VEGF moAbs. Also in this case, although the retrospective analysis extended to rare mutations of RAS, there was no significant difference between the two monoclonal antibodies in terms of OS (32.0 vs 31.2). These data suggest that both these are alternative treatments in this setting. The analysis of RR emphasized a difference in RR in favor of regimens containing the anti EGFR (68.6% vs 53.6%) [43, 44]. Finally, the randomized phase II PEAK trial, which compared FOLFOX+Panitumumab with FOLFOX+Bevacizumab in wild-type KRAS exon 2 untreated patients, showed an improvement of PFS and a trend towards improved OS in panitumumab-treated patients. However these results are limited by the small sample size [45, 46].

A recent meta-analysis evaluated data from nine trials as regards KRAS exon 2 wild-type tumors and other RAS mutations (in exons 3 and 4 of KRAS and exons 2, 3 and 4 of NRAS) as predictive factors of primary resistance. By pooled HRs no PFS or OS benefits were observed when anti-EGFR moAbs were delivered to patients with any RAS mutation. As a consequence all these RAS mutations are deemed as negative predictive factors [16].

In conclusion, the outcomes of all these analyses, which include rare RAS mutations, do not give oncologists a great deal of information on what is the best combination regimen for mCRC patients. In the absence of significant advantages in terms of OS, these new data regarding the response rate associated with the initial clinical evaluation of the patient may allow the construction of a treatment algorithm to guide the choice of a combination regimen in all RAS wild-type patients, with the addition of an anti-EGFR moAb in case of highly symptomatic patients who require a rapid reduction in tumor volume or, alternatively, of a regimen containing an anti-VEGF moAb, which also becomes the first choice of treatment in RAS mutated patients or carriers of a BRAF mutation [34].

## THE IMPACT OF OTHER PREDICTIVE BIOMARKERS

As reported above, RAS mutations represent the most important predictive biomarker of resistance to anti-EGFR therapy in mCRC, and the only one approved for clinical use. However mutant RAS only accounts for about 50% of non-responders to anti-EGFR therapy. Therefore additional predictive biomarkers are needed in order to better select those patients who may really gain benefit from anti-EGFR treatments, and sparing others from a futile treatment and related toxicities.

### BRAF mutations

BRAF is a proto-oncogene localized on chromosome 7 (7q34), encoding a serine-threonine protein kinase, and is a member of the RAF subfamily (together with the ARAF and RAF1 protein) which directly interacts with RAS. Mutations of B-RAF are reported in approximately 3-15% of mCRC and are mutually exclusive with RAS mutations [47, 48]. As in other malignancies, such as melanoma or NSCLC, the most common reported mutation is V600E, accounting for about 80% of all BRAF mutations in mCRC [49]. It consists of a substitution of valine with glutamate at codon 600 on exon 15, resulting in a constitutive activation of the RAF kinase and of the RAF–MEK–ERK downstream signaling pathway, responsible for both cancer cell proliferation and survival, independently of the EGFR blockade [50, 51]. Much evidence suggests that BRAF mutation is a strong negative prognostic factor in mCRC patients, regardless of treatments received [48, 52-58]. Although two meta-analyses have recently shown a negative predictive value of BRAF mutations for anti-EGFR treatment in mCRC [59, 60], their potential application for clinical practice is still debated. Retrospective studies have shown that the occurrence of a BRAF mutation is significantly associated with inferior outcomes in RAS WT, mCRC patients receiving anti-EGFR therapy as second or subsequent lines of treatment. Even if these data were not confirmed in the updated analysis of the study of Peeters et al. [61], BRAF mutation seems to be a negative predictor for anti-EGFR moAb treatment in the chemo-refractory setting [48, 62, 63]. On the other hand, unlike RAS mutations, the predictive value of B-RAF mutations in the first-line setting has not been fully demonstrated. Some retrospective studies have suggested that the BRAF mutation confers resistance to anti-EGFR therapy [64, 65], while a retrospective analysis of the COIN trial did not support the negative predictive role of the BRAF V600E mutation [66]. A pooled analysis including both CRYSTAL and OPUS trials retrospectively investigated the efficacy of Cetuximab plus chemotherapy in KRAS wild-type mCRC patients, according to BRAF mutational status. The results of such analyses have shown a significant benefit associated with the addition of anti-EGFR to chemotherapy in the KRAS/BRAF wild-type population, while the improvements in both PFS and OS among the 70 KRAS wild-type / BRAF mutated patients did not reach statistical significance, likely because of the small size of the B-RAF mutant population included in both the studies. Furthermore, B-RAF mutated patients treated with chemotherapy alone also reported worse outcomes compared with the B-RAF WT population, suggesting a predominant negative prognostic value of the B-RAF mutation [67]. The subgroup analysis of BRAF mutations in both the phase III PRIME study and FIRE-3 study, respectively comparing panitumumab plus FOLFOX versus FOLFOX and Cetuximab plus FOLFIRI vs Bevacizumab plus FOLFIRI as first-line treatment for mCRC, also did not confirm the predictive value of B-RAF mutations in this setting of patients [15, 68].

Recently, a prospective study evaluating the predictive significance of the BRAFV600E mutation in KRAS wild-type mCRC patients, in treatment with anti-EGFR therapies, showed that patients with BRAF mutated tumors have lower PFS and OS compared to patients with BRAF WT. Such survival differences were not statistically different in the first line-setting, while they reached a significant relevance in second or subsequent lines of treatment, according to previous evidence [69]. To date, the available data are insufficient to support the use of BRAF mutations as clinical predictive biomarkers for the upfront selection of mCRC patients, candidate to anti-EGFR therapy, and their low prevalence further limits their potential utility in clinical practice. However, another study presented at the 2014 ASCO meeting reported BRAF mutations in 11% of 37 mCRC patients with acquired resistance to cetuximab-based treatment, suggesting also a potential role in the development of resistance during anti-EGFR therapy [70]. BRAF mutations have also been detected noninvasively by ct-DNA analysis at the time of progression in mCRC patients who had previously responded to anti-EGFR therapy, supporting the idea that such a mutation alone, or together with other molecular alterations, such as RAS mutations, may sustain the emergence of acquired resistance during anti-EGFR treatments [17, 71].

### PIK3CA mutations

PIK3CA is a proto-oncogene localized on chromosome 3 (3q26.3), encoding the catalytic (p110) subunit of the class IA phosphatidylinositol3-kinases (PI3K). It is an intracellular lipidic kinase, recruited by the EGFR tyrosine-kinase domain, able to convert phosphatidylinositol-bisphosphate (PIP2) to phosphatidylinositol-triphosphate (PIP3), and ultimately responsible for the phosphorylation of AKT and the activation of the downstream AKT-mTOR signaling pathway, mediating both cell proliferation and survival [72]. Somatic mutations of the PIK3CA gene are reported in about 15-20% of CRC, and may co-exist with both RAS and BRAF mutations [62, 73]. More than 80% of PIK3CA mutations map to the exon 9 (60-65% of mutations) and exon 20 (20-25% of mutations) hot-spots [62, 74], leading to the constitutive activation of the PI3K catalytic (p110) subunit and the downstream AKT/mTOR signaling pathway, independently of EGFR blocking. Some retrospective studies have investigated the potential role of PIK3CA mutations as a predictor of resistance to anti-EGFR treatments in mCRC patients, with conflicting results. Sartore-Bianchi et al. showed that KRAS wild-type mCRC patients under anti-EGFR treatment whose tumors harbored PIK3CA mutations had significantly lower RR and PFS compared to a PIK3CA WT population. Moreover all responders were found to be PIK3CA wild-type [75]. In contrast, Prenen et al. did not support a significant difference in both RR and survival outcomes between PIK3CA mutated and WT mCRC patients treated with Cetuximab as monotherapy or in combination with irinotecan, in second or subsequent lines of treatment [74]. Finally, a large retrospective European analysis, involving 708 mCRC chemo-refractory patients, under anti-EGFR-treatment, showed that exon 20, but not exon 9, mutations of the PIK3CA gene were significantly associated with lower RR, PFS, and OS, compared to a WT population. Moreover exon 20 mutations were mutually exclusive with KRAS mutations, whereas exon 9 mutations were more often concomitant with KRAS mutated than KRAS wild-type CRC [62]. Such evidence allows to explain also the conflicting results of the two aforementioned studies, which were both characterized by a significant heterogeneity of the distribution of mutations in the two different exons in the included cohorts. Indeed the study by Sartore-Bianchi on PIK3CA mutated patients included respectively 73% exon 20 and 27% exon 9 mutations, compared to 13% and 78% exon 20 and 9 mutations respectively, in the study by Prenen et al. Overall these data suggest that PIK3CA exon 20 mutations may play a role as predictive biomarkers of resistance to anti-EGFR therapy in KRAS WT mCRC patients, as confirmed in two recent meta-analysis [60, 76]. However, because of its low frequency, further prospective studies are needed to support the clinical utility of PIK3CA analysis in the selection of those patients candidate to anti-EGFR therapy.

### PTEN loss

PTEN is a tumour suppressor gene, localized on chromosome 10 (10q23.3), encoding a phosphatase protein, which negatively regulates the PI3K-AKT signaling pathway. Loss of the PTEN protein through both genetic and epigenetic mechanisms is reported in about 20-40% of mCRC [77], resulting in a constitutive hyperactivation of the PI3K-AKT signaling pathway, responsible for tumor cell proliferation and survival [78]. Some studies have retrospectively investigated the predictive value of PTEN loss in mCRC patients under treatment with anti-EGFR therapy, reporting conflicting and inconclusive results. Loupakis et al. have shown that PTEN loss, detected by the IHC method, was associated with lower responses, compared with PTEN positive tumours of 102 chemo-refractory mCRC patients, treated with cetuximab + irinotecan. However such differences were significant in the metastatic lesions, but not in the primary tumours, suggesting that, unlike RAS mutations, PTEN expression may change over time [79]. Furthermore the analysis of survival outcomes, according to PTEN expression in metastatic lesions, has also shown a significant difference in PFS, in favor of the PTEN positive cohort, while a significant improvement in OS was observed only in PTEN positive, RAS wild-type patients. Another study by Sartore-Bianchi confirmed a significant association between PTEN loss and lack of benefit from anti-EGFR therapy in mCRC patients, both in terms of RR and PFS/OS. Finally a retrospective French study did not show any differences in terms of RR and PFS, according to PTEN expression, in a cohort of chemo-refractory, RAS wild-type mCRC patients [64]. However, a significantly lower OS was reported in the subgroup of PTEN negative patients, compared to those with PTEN positive tumors, suggesting a potential negative prognostic role of PTEN loss. Even if a recent meta-analysis confirmed that PTEN loss was significantly associated with lack of benefit to anti-EGFR therapy in RAS wild-type mCRC patients, the authors concluded that the predictive power of B-RAF and PIK3CA mutations were stronger than PTEN loss, not excluding that some patients with a single alteration could still gain benefit from anti-EGFR treatment [60]. Therefore, larger and prospective clinical trials, including a standardized method to detect PTEN expression, are required to establish its potential role as a predictive biomarker in RAS wild-type mCRC patients candidate to anti-EGFR-therapy.

### AREG and EREG expression

The expression of the EGFR ligands amphiregulin (AREG) and epiregulin (EREG) has been suggested as a possible mechanism that could influence the response to the anti-EGFR moAbs, cetuximab and panitumumab, in mCRC patients. A high concentration of these ligands is supposed to be related with a more aggressive tumor growth, but the rates of KRAS mutations are lower in tumors overexpressing EGFR ligands [80]. A retrospective study evaluated AREG and EREG expression in 220 mCRC patients enrolled in clinical trials (EVEREST, BOND, SALVAGE, and BABEL) [11, 81, 82]. The analyses were performed on formalin-fixed paraffin-embedded primary tumor samples through RNA extraction and real time PCR. High levels of EREG have been associated with a higher probability of objective responses and the majority of patients with high levels of EREG belonged to the group with complete and partial responses. Moreover, high levels of EREG expression in KRAS wild-type patients treated with irinotecan and cetuximab conferred a benefit in PFS in comparison with those with low ligand expression whose behavior was similar to patients with KRAS mutant tumors [83]. A recent study presented by J.F. Selingman et al. at the 2014 ASCO annual meeting evaluated the correlation between the expression levels of both AREG and EREG mRNAs in 323 RAS wild-type patients treated with irinotecan as second line therapy, with or without Panitumumab and enrolled in the randomized PICCOLO trial [63]. The results showed that the high level of expression of the mRNAs of both ligands had a predictive value for the RAS wild-type population demonstrating a benefit in PFS for those patients treated with irinotecan and Panitumumab versus irinotecan alone (mPFS 8.3 vs 4.4 months; HR: 0.62; 95% CI 0.49 – 0.78; p< .001), while for RAS wild-type patients with low AREG and EREG expression, no benefit was obtained. No prognostic significance was found in terms of PFS or OS [84]. These data indicate that the evaluation of the expression levels of EGFR ligands could be a promising path towards a more accurate selection of RAS wild-type patients to treat with anti-EGFR drugs, but more consistent evidence is needed to make this hypothesis part of daily practice.

### HER-2 amplification

HER2 is a receptor that belongs to the ErbB family and whose activation does not require the presence of a ligand. Indeed it depends on the heterodimerization of HER2 with other similar receptors of the family. A high level of activation of HER2 can also be promoted by gene amplification and subsequent HER2 overexpression [85]. Preclinical evidence suggested a possible role of the heterodimers of EGFR with other members of the HER family, such as HER2 and HER3, in affecting the efficacy of anti-EGFR based therapies. Two studies have demonstrated a correlation between the amplification of HER2 and the acquired resistance of mCRC to cetuximab therapy (see below) [86, 87]. Recent retrospective data confirm that the analysis by FISH of the HER2 amplification status could represent a promising way to better identify subgroups of mCRC patients for treatment with anti-EGFR drugs. Martin et al. evaluated HER2 gene status in 170 KRAS wild-type mCRC patients treated with cetuximab or panitumumab identifying three profiles: a) patients with no or slight HER2 amplification (35%); b) patients with HER2 amplification in minor clones or with increased HER2 gene copy number due to polysomy (HER2-CNG) (61%); c) patients with HER2 amplification in all cells. The worst outcome was seen in the group of patients with amplification in all cells, while intermediate outcomes were seen for patients with no amplification. Interestingly the best outcomes were seen in the group of patients with amplification in minor clones or polysomy. Authors explain these different results supposing that tumors in the group with HER2 amplification in a minority of the cells or with HER2 polysomy may have a different pathogenesis linked to a general chromosome instability. In the group with HER2 amplification in all cells however, HER2 activation can bypass the blockade of EGFR mediated by panitumumab and cetuximab, inducing a strong resistance to these moAbs but placing the biological basis for anti-HER2 therapy in this group of patients. The lack of efficacy of anti-EGFR treatment for the patients with no amplification of HER2 has been related to kariotypic heterogeneity [88]. In a recently published work, Cushman et al. investigated the expression of EGFR axis-related genes, analysing 103 tumor samples from the CALGB 80203 trial to find prognostic or predictive markers for patients with mCRC and treated with cetuximab and chemotherapy as first line treatment: the expression levels of 14 EGFR-related genes were correlated with clinical outcomes (OS and PFS). Surprisingly, high mRNA levels of HER2 (HR=0.64) and epiregulin (HR=0.89) showed a prognostic association with longer PFS for all patients while a positive trend toward better OS was found for the same genes in the overall population [89, 90]. Although these data are preliminary and partially conflicting, they confirm a potential role of HER2 overexpression in guiding the response of tumors to anti-EGFR treatments. At the moment, however, such evidence does not allow definitive conclusions and prospective studies are needed for this purpose.

## PRECLINICAL AND TRANSLATIONAL FINDINGS ABOUT RAS-RELATED ACQUIRED RESISTANCE

Since anti-EGFR moAbs were introduced into clinical practice for the treatment of mCRC, primary resistance has been studied to select those patients with higher probability of benefit. Concomitantly the mechanisms of acquired resistance have been investigated as cancer progression steadily develops during treatment by these targeted agents.

Among the first investigations on acquired resistance to cetuximab, Wheeler et al. examined the activity of 42 receptor tyrosine kinases (RTK) in cetuximab-resistant cell lines. They found high activity of EGFR, HER2, HER3 and cMET. This phenomenon seems to be mediated by an overexpression of EGFR as a consequence of disregulated internalization and degradation of EGFR, which subsequently heterodimerizes with the other RTKs and transactivates them. However HER3 activation appears to be more crucial than the other RTKs in inducing acquired resistance to cetuximab [91].

Cetuximab-resistant clones have a marked nuclear expression of EGFR. This EGFR in the nuclear compartment works as a transcription factor for cyclin D1, iNOS, B-myb and Aurora kinase A, and phosphorylates and stabilizes PCNA. The accumulation of EGFR in the nucleus is favoured by several EGFR ligands, such as EGF, HB-EGF, AR and β-cellulin. Src family kinase (SFK) is a mediator of EGFR translocation, as its inhibition by dasatinib may restore sensitivity to cetuximab [92].

Further *in vitro* studies on anti-EGFR-resistant cells showed higher levels of phosphorylated EGFR, MAPK, AKT and STAT3, which were associated with reduced apoptosis [93]. Some preclinical studies on resistant cells in xenograft models discovered a role of angiogenesis dysregulation. Angiogenesis is an important determinant of tumor progression, therefore new therapeutic approaches are necessary to control angiogenic spread [94]. These angiogenic mechanisms involved in resistance to anti-EGFR agents include increased expression of VEGF and other VEGFR-1 ligands. Despite this intriguing preclinical evidence, phase III randomized trials did not find benefit from combined inhibition of EGFR and VEGF pathways [95-98].

The role of KRAS mutations in acquired resistance to anti-EGFR moAbs was first reported by Bouchahda et al. [99]. These authors published a case report of a mCRC patient with a KRAS wild-type primary tumor and synchronous metastases evaluated before beginning treatment with cetuximab. However, after initial significant tumor shrinkage on cetuximab and subsequent progression, a segment hepatectomy allowed to evaluate the mutational status of a metachronous liver metastasis. They detected both codon 12 and 13 KRAS mutations. These mutations are known to be very early events in colorectal carcinogenesis. This observation is corroborated by the findings of high concordance, about 90%, in primary tumors and in metastases [79, 100-102].

For this reason it was argued that the onset of new KRAS mutations could be a consequence of clonal selection of cells early mutated in the KRAS gene. This selection is the effect of selective pressure by anti-EGFR moAbs. An alternative explanation is represented by the development of novel spontaneous mutations, maybe favoured by cytotoxic therapy. The intratumor heterogeneity of the cancer cell population is the better background for the genesis of potential drug-resistant metastatic clones and it may also explain why the mutations in these clones could be missed in DNA detection in tumor samples[103, 104].

Further genomic alterations have been implicated in acquired resistance to anti-EGFR moAbs in preclinical studies [105]. Aberrant ERBB2 signalling, by ERBB2 amplification or heregulin production, seems to be responsible for both intrinsic and acquired resistance in cetuximab-treated mCRC patients. This ERBB2 signalling impairment represents the activation of a bypass signalling pathway. The aberrant activation of the ERBB2 gene leads to persistent ERK1/2 signalling during treatment with cetuximab, and subsequently the development of acquired resistance to this agent. Of note, breast cancer cells treated with the anti-ERBB2 moAb trastuzumab have an activation of EGFR signalling as a mechanism of acquired resistance. However this phenomenon is not observed for tyrosine kinase inhibitors (TKI), which can inhibit both EGFR and ERBB2 at clinically achievable concentrations [86, 87, 106, 107].

The amplification of other genes was also observed as a mechanism of *de novo* and acquired resistance. The amplification of the KRAS gene is mutually exclusive with KRAS or BRAF mutations. It induces overexpression of the KRAS wild-type protein with changes of gene expression similar to those caused by mutant KRAS variants. However this genomic alteration was reported in a small proportion of patients treated with anti-EGFR moAbs, around 4%. Besides, KRAS amplification has been shown to have a deterministic effect on anti-EGFR resistance in *in vitro* studies. Indeed its occurrence in sensitive cells favours resistance and its silencing restores sensitivity [108].

Similarly MET amplification was found at high levels in patients who progressed during treatment with anti-EGFR therapy. In the same study other gene copy number variations were not found, while the other resistance-inducing oncogenes remained non-mutated. Interestingly MET amplification can be detected in circulating DNA over time. MET amplification was found at low levels in tumor tissues before treatment. As a consequence it could represent theoretically a mechanism for *de novo* resistance. But the small amount of MET-amplified cells can be expanded by the selective pressure of anti-EGFR moAbs and they can induce acquired resistance, as they become the leading tumor cell population. This phenomenon has highly relevant therapeutic implications, since MET is an actionable target and MET inhibitors, such as crizotinib, are under investigation [109].

A missense mutation in the EGFR extracellular domain (S492R) was detected by sequencing analysis. This mutation hinders cetuximab binding and it resulted in the retention of EGFR activity during exposure to cetuximab but not panitumumab. It was not detected in tumor samples from untreated mCRC patients [110, 111].

A cross talk between EGFR and MET has been shown. EGFR immunoprecipitated together with MET in cetuximab-resistant colorectal cancer cells, and this interaction was also observed in parental cells by TGFα stimulation. This interaction between EGFR and MET induces the activation of MET as a consequence of a stimulation with ligands binding both receptors and has a synergistic effect on the downstream effector of respective pathways. Moreover TGFα plasma levels were higher during treatment with cetuximab, even though they do not influence tumor response. Accordingly, high TGFα mRNA levels were significantly increased in patients progressing during cetuximab treatment. Therefore the overexpression of TGFα induces the interaction of EGFR and MET, with subsequent phosphorylation and activation of MET and its effectors. This mechanism could explain the development of resistance to anti-EGFR agents and it suggests to overcome this resistance by the combined inhibition of EGFR and MET. Conversely high expression levels of AREG and EREG ligands are related to a better response to cetuximab [112-114].

More recently the paracrine secretion of EGFR ligands has been implicated in the acquired resistance to cetuximab. Some authors have suggested a “paracrine *in trans* protection of sensitive cells by their mutated derivatives”. According to this explanation, sensitive RAS wild-type cells and resistant RAS-mutated cells cooperate to develop the acquired resistance [115].

The downstream molecules in the EGFR pathways have also been studied to explain the mechanisms of acquired resistance to anti-EGFR moAbs. In addition to the mutations in the genes involved in the same pathway and the pathways cross-talking (KRAS, BRAF, NRAS, MET and HER2), a sustained activation of MEK and ERK in terms of constitutive phosphorylation has been implicated in acquired resistance. However the blockade of MEK is not sufficient to inhibit the proliferation of resistant cells. Only the concomitant blockade of EGFR and MEK achieves a reversion of secondary resistance. This evidence has been explained by the transient ERK inactivation upon MEK inhibition, which leads to the phosphorylation of EGFR. However a mechanism of this EGFR feedback activation has not yet been found [71].

Accordingly, in another *in vitro* study the acquired resistance was defined by the lack of blockade of MAPK and AKT activation in spite of EGFR inhibition. In this study the combined treatment with cetuximab and a MEK1/2 inhibitor has a synergistic effect, as can be verified by the inhibition of activated pAKT and pMAPK. This inhibition leads to cell apoptosis as confirmed by increased levels of cleaved PARP and caspase-3 activation. MEK1/2 silencing restores the ability of cetuximab to inhibit MAPK and cell proliferation [116].

All these studies led to the identification of possible mechanisms to explain the onset of acquired resistance to anti-EGFR moAbs in mCRC patients. Only a small amount of genes appear to confer resistance. And this observation is encouraging for attempting to overcome the resistance. The new approach of liquid biopsy by cell-free circulating tumor DNA allowed to monitor the appearance of mutations of these genes in wild-type patients [17].

As a consequence of these findings a question arises: how could gene mutations responsible for acquired resistence to anti-EGFR moAbs emerge? Some studies could provide an answer to this question. The study by Diaz et al. provided further data about gene mutations in circulating DNA. The authors developed a mathematical model which led to the conclusion that these mutations were present in expanded subclones prior to the initiation of the anti-EGFR moAbs. The results of this analysis agree with the in vitro study by Misale S et al., who continuously treated colon cancer cell lines with cetuximab leading to the emergence of resistant variants. The molecular alterations, KRAS mutations and amplification identified in resistant cells were also detected in a minority population of parental cells. These results suggest that the change from sensitive to resistant phenotype could derive from selection of pre-existing resistant clones. However a further experiment on sensitive cells, which were confirmed to be wild-type by high-sensitivity analyses, showed the appearance of KRAS mutation upon treatment with increasing concentrations of cetuximab. Therefore the resistance to anti-EGFR moAbs may also emerge by ongoing mutagenesis. Further preclinical studies are needed to explain these phenomena, but all these findings helped to propose new treatment strategies to overcome or prevent resistance to anti-EGFR moAbs in mCRC patients [26, 117, 118].

It is usually thought that anti-EGFR moAbs work by avoiding the activation of EGFR pathway by its ligands. A significant apoptosis of tumor cells was not observed by Cetuximab. Tyrosine kinase inhibitors of EGFR have not shown efficacy in colon cancer patients yet. Some researchers hypothesized a role by antibody-dependent cellular cytotoxicity (ADCC) [119, 120]. The binding of the Fc region of a moAb to a Fc receptor expressed with different patterns by cells of the innate immune system, namely monocytes, macrophages, granulocytes and natural killer cells (NK). Cetuximab was able to mediate NK-dependent ADCC in vitro, since NK cells are the main mediators of the ADCC-dependent therapeutic effects by cetuximab [121]. Instead, panitumumab is less effective in inducing NK-dependent ADCC, maybe because IgG2 immunoglobulins have a reduced avidity for Fc receptor in comparison with IgG1. It has been supposed that the entity of ADCC in cancer patients treated with cetuximab may help to predict tumor response. In colorectal cancer cells KRAS mutations may interfere with ADCC activity. Besides cancer patients may experience a lower induction of ADCC by cetuximab in comparison with healthy subjects as related to potential immunosuppression in cancer patients [122]. Myeloid-derived suppressor cells and other inflammatory cells could explain this limitation of cetuximab-related ADCC.

## CONCLUSIONS

The introduction of anti-EGFR moAbs in combination with chemotherapy regimens for mCRC patients improved OS and quality of life. Nowadays these agents are the standard treatment for these patients. However several clinical studies have highlighted the predictive role of gene alterations involved in the EGFR pathway. For each mCRC patient the analysis of these biomarkers in tissue biopsy is mandatory to decide the proper treatment. Until now many genomic alterations have been identified. Among these biomarkers RAS mutations in exon 2 and 3 are the most clearly defined for clinical practice. The data for the other biomarkers, including BRAF mutations, PIK3CA mutations, PTEN loss, AREG and EREG expression and HER-2 amplification, are still inconclusive or incomplete. Further findings are emerging regarding the role of these biomarkers as predictive for acquired resistance. Liquid biopsy will help to identify and monitor the biomarkers for both primary and acquired resistance. The application of this new method will be granted by the validation of high-sensitivity techniques for the isolation and detection of somatic mutations in cell-free circulating tumor DNA. Besides, it will allow to identify various mutations at the same time. This opportunity will speed up the process of patient selection for anti-EGFR moAbs.
